# Phase 2 study of Wee1 inhibitor adavosertib in recurrent uterine carcinosarcoma

**DOI:** 10.1016/j.gore.2025.101796

**Published:** 2025-06-24

**Authors:** Stephanie Cham, Niya Xiong, Nabihah Tayob, Carolyn Krasner, Alexi A. Wright, Elizabeth K. Lee, Hannah Sawyer, Cara Mathews, Panagiotis A. Konstantinopoulos, Ursula A. Matulonis, Joyce F. Liu

**Affiliations:** a Department of Obstetrics and Gynecology & Reproductive Sciences, University of California San Francisco, San Francisco, CA, United States of America; b Department of Data Science, Dana-Farber Cancer Institute, Boston, MA, United States of America; c Department of Medical Oncology, Dana-Farber Cancer Institute, Boston, MA, United States of America; d Women & Infants Program Hospital Program in Women’s Oncology, Alpert Medical School of Brown University, Providence, RI, United States of America

**Keywords:** Uterine carcinosarcoma, Wee1 inhibitor, TP53 mutation, Phase 2 trial, Next generation sequencing

## Abstract

**Purpose:**

Uterine carcinosarcoma (UCS) is a rare but aggressive tumor with high rates of recurrence and poor prognosis, and novel therapies are urgently needed. P53 mutations are identified in over 90% of cases, and other cell cycle alterations are commonly found indicating potential vulnerability to Wee1 kinase inhibition. The purpose of this study was to determine the activity and safety of adavosertib, a Wee1 inhibitor, in recurrent or persistent UCS.

**Patients and methods:**

This was a phase II single-institution study of patients with persistent or recurrent UCS. Eligible patients had a confirmed *TP53* alteration, RECIST measurable disease, and prior treatment with platinum based systemic therapy. Patients were treated with adavosertib at a starting dose of 300 mg orally once daily days 1–5 and 8–12 of a 21 day cycle until progression. The co-primary endpoints were objective response rate (ORR) and rate of progression-free survival (PFS) at 6 months. Molecular alterations were identified by targeted next generation sequencing where available.

**Results:**

9 patients enrolled prior to drug discontinuation by the sponsor. ORR was 22.2 % (95 % CI 2.8–60 %) with 2 partial responses. 3 patients (33.3 %) had stable disease. Median PFS was 2.7 months (95 % CI 0.9 − not reached). Treatment-related adverse events (all grades) occurred in 8 patients (88.9 %), most commonly diarrhea (77.8 %) and fatigue (66.7 %).

**Conclusion:**

In this phase II trial of 9 patients with *TP53*-mutated UCS, adavosertib demonstrated limited activity. However, future studies of molecular alterations and combinatorial strategies continue to be of interest in UCS with limited treatment options.

## Highlights

• In a phase 2 single-arm study (N = 9) of uterine carcinosarcoma with confirmed *TP53* alteration, ORR to adavosertib was 22.2 %.
• 2 patients (22.2 %) had a partial response, 3 (33.3%) had stable disease.
• Median progression free survival time was 2.7 months (95 % CI 0.9 to not reached).
• Treatment-related adverse events occurred in 8 patients (88.9 %) most commonly diarrhea and fatigue.
• Accrual was terminated early due to discontinuation of development of adavosertib.

## 1 Introduction

Uterine carcinosarcoma (UCS) is a rare neoplasm representing 5 % of uterine tumors but disproportionately accounts for 15 % of all deaths caused by uterine corpus malignancy (Cantrell et al., 2015). UCS has a biphasic histology composed of both epithelial and sarcomatous/mesenchymal elements, and while the origin has been the subject of debate, current molecular and genetic studies support a monoclonal origin where tumors are thought to arise from high grade carcinoma followed by sarcomatous differentiation (or an epithelial to mesenchymal transition). UCS is considered an aggressive tumor with upwards of 60 % of UCS patients present with extra-uterine disease (Kernochan and Garcia, 2009). Overall, UCS carries a poor prognosis with a recurrence rate of 37 % in stage I disease and up to 80 % in stage IV disease, with dismal 5 year overall survival rates of 59–65 % for patients diagnosed with stage I disease and 9–26 % for those diagnosed at stage IV (Cantrell et al., 2015). Importantly, UCS, among other high grade endometrial cancer subtypes, has been shown to have a 2–4 fold increase incidence in non-Hispanic black populations and likely contributes to the relative racial ethnic health disparities seen in endometrial cancer (Winkler et al., 2024, Abel et al., 2021).

At present the standard treatment for UCS includes hysterectomy with staging with cytoreduction for advanced disease and chemotherapy. Prospective phase 3 trials to determine the optimal adjuvant therapy for UCS have primarily included radiation or comparisons of chemotherapy regimens with little yield in improved outcomes. A phase 3 trial in chemotherapy naïve patients with recurrent or persistent UCS demonstrated that a platinum/taxane based regimen was not inferior in terms of progression free survival and overall survival when compared to a platinum/ifosfamide combination, and carboplatin/paclitaxel has thus become the general standard of care first line systemic therapy (Powell et al., 2022). However, active therapies beyond first-line therapy are limited. While new Her2 targeted therapies have shown encouraging clinical activity, given the high rate of recurrence and poor prognosis of this disease, novel therapies continue to be urgently needed (Nishikawa et al., 2023, Meric-Bernstam et al., 2024).

A small number of studies examining the landscape of genomic alterations in UCS have identified *TP53* mutations and alterations, amplifications, or loss of function in cell-cycle related genes as a common molecular alterations, making Wee1 kinase inhibition a potential therapeutic strategy (Toboni et al., 2021). In the largest molecular characterization analysis of UCS which included 57 primary tumors, *TP53* mutations were identified in 91 % of patients. UCS tumors also frequently harbor alterations in *FBXW7*, *PPP2R1A*, *KRAS*, *RB1* and *PIK3CA* and cell-cycle alterations with potentially clinically relevant implications were identified in 22.8 % of patients including alterations in *CCND1*, *CDKN2B*, *CCNE1*, and *CDKN2A* (Cherniack et al., 2017). In another study examining clinical correlates of somatic mutations in UCS, *TP53* mutation was associated with markedly decreased overall survival (HR 55, p < 0.001) (Growdon et al., 2011). Given the role of Wee1 in regulating CDK1/2 and the G2/M checkpoint cells that have alterations in cell cycle regulation in combination with either the presence of homologous recombination deficiency or an oncogenic driver (both of which can increase replication stress) may display increased susceptibility to Wee1 inhibition (Bridges et al., 2011, Gourley et al., 2019).

Studies of Wee1 inhibition in uterine serous carcinoma, which shares many of the same molecular alterations as UCS, have reported evidence of clinical activity with phase II studies show objective response rates (ORR) of 25–29 % (Liu et al., 2021, Funda Meric-Bernstam et al., 2022, Liu et al., 2023). Given the molecular similarities between uterine serous carcinomas and UCS, we hypothesized that Wee1-directed therapy may also be active in UCS. The purpose of this study was therefore to evaluate the clinical activity and safety of adavosertib, a Wee1 inhibitor, in recurrent or persistent UCS in a single-arm phase II trial.

## 2 Methods

### 2.1 Patient selection

Eligible participants had histologically or cytologically confirmed recurrent UCS. Additionally, tumors also needed to have: (1) presence of a p53 alteration (by either immunohistochemistry staining or next generation sequencing), (2) metastatic or extra-uterine component confirmed on pathology to be from the carcinoma component of the disease. For patients without confirmation that the metastatic or extrauterine component of disease was of carcinoma histology, they could be considered for trial enrollment after discussion with the principal investigator if the primary disease was predominantly composed of carcinoma histology, biopsiable disease was present, and the patient as amenable to pre- and on-treatment biopsies. Patients had to have measurable disease per Response Evaluation Criteria in Solid Tumors (RECIST) 1.1 criteria and have had at least one prior platinum-based systemic chemotherapy. Patients with MSI-high or MMR-deficient tumors needed to have received prior PD1 or PD-L1-directed therapy or be considered not a candidate for immune checkpoint therapy. There were no restrictions on the number of prior lines received and prior radiation treatment was allowed. Patients were required to be 18 years or older, ECOG performance status of ≤1, have adequate organ and bone marrow function defined by absolute neutrophil count of ≥1500/mcL, haemoglobin ≥9 g/dL, and platelets ≥100,000/mcL. Patients had to be able to swallow oral medications and could not have an existing gastrostomy tube, be receiving total parenteral nutrition, or be dependent on intravenous fluid support.

### 2.2 Study design

This is a multi-cohort single arm study phase II study conducted at Dana-Farber Cancer Institute in Boston, MA. The study was reviewed and approved by the Dana-Farber and Harvard Cancer Center Institutional Review Board and conducted in accordance with ethical principles per the Declaration of Helsinki. The trial was registered on Clinicaltrials.gov (NCT03668340) and here we report one of the three cohorts in the trial design. Details of the uterine serous carcinoma cohort are reported elsewhere (Liu et al., 2021, Liu et al., 2023). All patients were provided informed consent. Participants in the study received adavosertib monotherapy starting at the established recommended phase 2 dosing of 300 mg orally once a day on days 1 through 5 and 8 through 12 of a 21 day cycle until progression or unacceptable toxicity. Participants were followed for 30 days after study drug discontinuation.

### 2.3 Safety and assessments

Physical exam and in-person patient assessment were performed on days 1 and 8 of the first two cycles of treatment and day 1 on all subsequent cycles. Laboratory studies, including a complete blood count with differential and serum chemistries were performed weekly during the first two cycles and subsequently on days 1 and 8 of each following cycle. AEs were graded according to the National Cancer Institute Common Terminology Criteria for Adverse Events (CTCAE) version 5.0. Radiologic disease assessments were performed at baseline and every two cycles for the first six cycles; thereafter, the interval between imaging assessments could be extended to every three cycles per the treating investigator’s discretion. Tumor response was evaluated by RECIST 1.1 (Eisenhauer et al., 2009).

### 2.4 Statistical analyses

This cohort used a single-stage study design, with a planned enrollment of 20 patients. The co-primary endpoints were to assess the activity of adavosertib as measured by ORR by RECIST 1.1 criteria and the rate of progression-free survival at 6 months (PFS6). For the co-primary endpoints the null hypothesis for the ORR was 5 % and for PFS6 was 25 % with an alternative hypothesis of 30 % ORR and 52 % PFS6, assuming a type 1 error of 0.057 and power of 0.89 for ORR and 0.80 for PFS6. With 20 patients enrolled, we would reject the null hypothesis if 4 treated patients experienced an OR or 9 patients were progression-free at 6 months and adavosertib would be considered worthy of further study in this patient population. The trial closed early to accrual when clinical development of adavosertib was discontinued by AstraZeneca.

Secondary objectives included assessment of the clinical benefit rate (CBR) as defined as the objective response rate plus stable disease for six months and assessment of the safety profile by reported AEs. Exploratory objectives included querying of molecular alterations to explore whether specific molecular alterations correlate with the activity of adavosertib in this patient population. For both binary end points, the exact 95 % CIs were estimated. Progression-free survival (PFS) was estimated by Kaplan-Meier curves. Statistical analyses were performed with R version 3.6.2.

### 2.5 Molecular analyses

Molecular alterations present in patient tumors were obtained from chart abstraction of clinically available testing results. Seven of nine patients had available targeted next generation sequencing results (six of these were evaluated using an in-house OncoPanel platform, which surveys exonic DNA sequences of 447 genes as previously described (Garcia et al., 2017), and the other patient had FoundationOne CDx platform (Foundation Medicine, Cambridge, MA). No formal statistical analyses were performed; the presence of molecular alterations was illustrated descriptively using an OncoPrint (generated by cBioPortal OncoPrinter) (Gao et al., 2013, Cerami et al., 2012).

## 3 Results

### 3.1 Patient characteristics

Between June 02, 2020 and December 06, 2022, 9 patients were enrolled in the study. Accrual was terminated prematurely when clinical development of adavosertib was discontinued. All patients were considered evaluable for analysis. Patient characteristics can be seen in Table 1. The median age was 69.7 years (range 58.7–79.5). The median number of prior therapies was 3. At the time of the data cut off on May 16, 2023, all follow-up had been completed, with 7 of the patients having completed 30-day follow-up per protocol, and 2 additional patients having withdrawn consent from further follow-up. The median follow-up time at the time of analysis was 3.7 months (IQR 2–5.4).

**Table 1:** Patient characteristics.

Characteristic	Overall (N = 9)
Median age (range)	69.7 (58.7–70.5)
Race, self-reported (%)	
Non-Hispanic White	7 (77.8)
Non-Hispanic Black or African American	2 (22.2)
ECOG PS (%)	
0	3 (33.3)
1	6 (66.7)
Stage at diagnosis (%)	
I	1 (11.1)
II	1 (11.1)
III	3 (33.3)
IV	4 (44.4)
Stage at treatment (%)	
Recurrent	8 (88.9 %)
Persistent	1 (11.1 %)
Histology (%)	
Well differentiated	1 (11.1)
Poorly differentiated	8 (88.9)
Method of TP53 evaluation	
IHC	3 (33.3 %)
NGS	6 (66.7 %)
Alterations found on NGS	
TP53 H179N	1 (16.7 %)
TP53 R248W	1 (16.7 %)
TP53 R273C	1 (16.7 %)
TP53 R337L	1 (16.7 %)
TP53 S241F	1 (16.7 %)
TP53 V274A	1 (16.7 %)
Her2 IHC expression (%)	
0	2 (22.2)
1+	2 (22.2)
2+	0 (0)
3+	1 (11.1)
Not available	4 (44.4)
Prior chemotherapy lines, median (range)	3 (1–6)
Prior chemotherapy types received, N (%)	
Carboplatin and paclitaxel	9 (100)
Weekly paclitaxel	2 (22.2)
Liposomal doxorubicin	2 (22.2)
Nab-paclitaxel	2 (22.2)
Lenvatinib/pembrolizumab	2 (22.2)
Cyclophosphamide/docetaxel	1 (11.1)
Cisplatin/gemcitabine	1 (11.1)
Bevacizumab	1 (11.1)
Everolimus	1 (11.1)
ONC-201	1 (11.1)
Pazopanib	1 (11.1)
Trastuzumab	1 (11.1)
Trastuzumab deruxtecan	1 (11.1)
Olaparib	1 (11.1)
Prior radiation, N (%)	
Yes	4 (44.4); 3 pelvic IMRT, 1 vaginal apex
No	5 (55.6)

### 3.2 Clinical activity

**Fig. 1:**
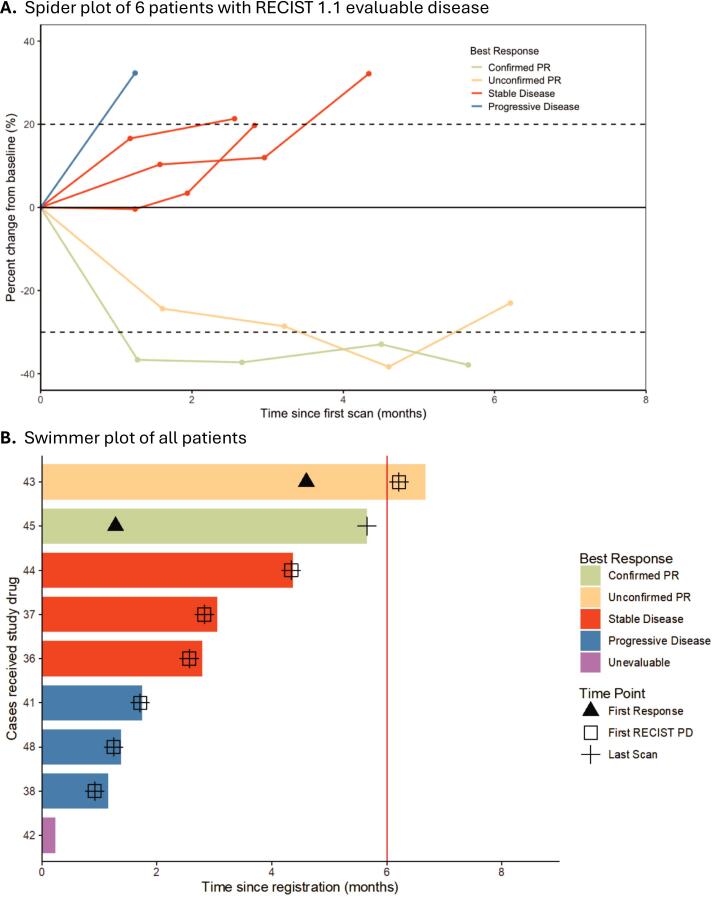
Clinical benefit experienced by patients on study as shown by (A) spider plot and (B) swimmer plot.

In the 9 enrolled patients, 2 patients (22.2 %; 95 % CI 2.8–60 %) had a partial response (1 confirmed and 1 unconfirmed). 3 patients (33.3 %) had stable disease and 3 (33.3 %) had progressive disease. 1 patient (11.1 %) withdrew prior to their first scan and was considered unevaluable for response. The overall response rate based on the 8 evaluable patients was 22.2 % (95 % CI 2.8–60 %). A spider plot of the observed clinical activity in the six patients with evaluable target lesions on at least one follow-up RECIST assessment is shown in Fig. 1**A**. The 1 unconfirmed PR was due to one scan showing a PR with subsequent follow-up scan not meeting criteria for a PR. 3 patients are not shown on the spider plot as 1 patient was unevaluable due to withdrawing prior to first scan, 2 patients showed progression in non-index lesions. Median PFS was 2.7 months (95 % CI 0.9 to not reached). One patient was alive and progression-free at 6 months with a partial response to treatment with radiographic response, as shown in the swimmer plot in Fig. 1**B**. One patient discontinued treatment for concerns of clinical disease progression just before 6 months. CBR was unable to be calculated due to the limited number of patients reaching the six month assessment mark.

### 3.3 Safety

Treatment-related AEs (TRAEs) occurred in 8 (88.9 %) patients. The most common were gastrointestinal disorders including diarrhea (77.8 %), nausea (66.7 %), and abdominal pain (33.3 %). Fatigue was commonly reported (66.7 %). Hematologic AEs were common including anemia (44.4 %), thrombocytopenia (33.3 %), and neutropenia (33.3 %). Abnormal laboratory findings were noted with almost all being grade 1 including acute kidney injury (11.1 %), hyponatremia (11.1 %), elevated AST levels (22.2 %) and elevated ALT levels (11.1 %). The most common Grade 3 or higher TRAEs included diarrhea (33.3 %), fatigue (22.2 %), anemia (22.2 %), and platelet count decreased (22.2 %). All TRAEs are listed in Table 2 and a list of treatment-emergent AEs is available in Supplemental Index 1. Five of the 9 patients (55.5 %) required dose modification with 33.3 % requiring one and 22.2 % requiring two dose reductions. Eight (88.9 %) required a dose to be held. No patients discontinued the study due to TRAEs. No grade 5 AEs occurred.

**Table 2:** Treatment-related adverse events.

Adverse events	Grade 1	Grade 2	Grade 3	Grade 4	All grade
Any	0(0 %)	3(33 %)	3(33 %)	2(22 %)	8(89 %)
Diarrhea	1(11 %)	3(33 %)	3(33 %)	0(0 %)	7(77 %)
Fatigue	0(0 %)	4(44 %)	2(22 %)	0(0 %)	6(66 %)
Nausea	3(33 %)	2(22 %)	1(11 %)	0(0 %)	6(66 %)
Anemia	0(0 %)	2(22 %)	2(22 %)	0(0 %)	4(44 %)
Platelet count decreased	1(11 %)	0(0 %)	1(11 %)	1(11 %)	3(33 %)
Neutrophil count decreased	1(11 %)	1(11 %)	0(0 %)	1(11 %)	3(33 %)
Abdominal pain	1(11 %)	2(22 %)	0(0 %)	0(0 %)	3(33 %)
Aspartate aminotransferase increased	2(22 %)	0(0 %)	0(0 %)	0(0 %)	2(22 %)
Anorexia	2(22 %)	0(0 %)	0(0 %)	0(0 %)	2(22 %)
Thromboembolic event	0(0 %)	0(0 %)	1(11 %)	0(0 %)	1(11 %)
Alanine aminotransferase increased	0(0 %)	1(11 %)	0(0 %)	0(0 %)	1(11 %)
Hypoalbuminemia	0(0 %)	1(11 %)	0(0 %)	0(0 %)	1(11 %)
Bloating	1(11 %)	0(0 %)	0(0 %)	0(0 %)	1(11 %)
Gastrointestinal disorders − Other, specify	1(11 %)	0(0 %)	0(0 %)	0(0 %)	1(11 %)
Oral pain	1(11 %)	0(0 %)	0(0 %)	0(0 %)	1(11 %)
Vomiting	1(11 %)	0(0 %)	0(0 %)	0(0 %)	1(11 %)
Alkaline phosphatase increased	1(11 %)	0(0 %)	0(0 %)	0(0 %)	1(11 %)
Creatinine increased	1(11 %)	0(0 %)	0(0 %)	0(0 %)	1(11 %)
Hyponatremia	1(11 %)	0(0 %)	0(0 %)	0(0 %)	1(11 %)
Flank pain	1(11 %)	0(0 %)	0(0 %)	0(0 %)	1(11 %)
Generalized muscle weakness	1(11 %)	0(0 %)	0(0 %)	0(0 %)	1(11 %)
Dizziness	1(11 %)	0(0 %)	0(0 %)	0(0 %)	1(11 %)
Dysgeusia	1(11 %)	0(0 %)	0(0 %)	0(0 %)	1(11 %)
Dyspnea	1(11 %)	0(0 %)	0(0 %)	0(0 %)	1(11 %)

### 3.4 Biomarker analyses

**Fig. 2:**
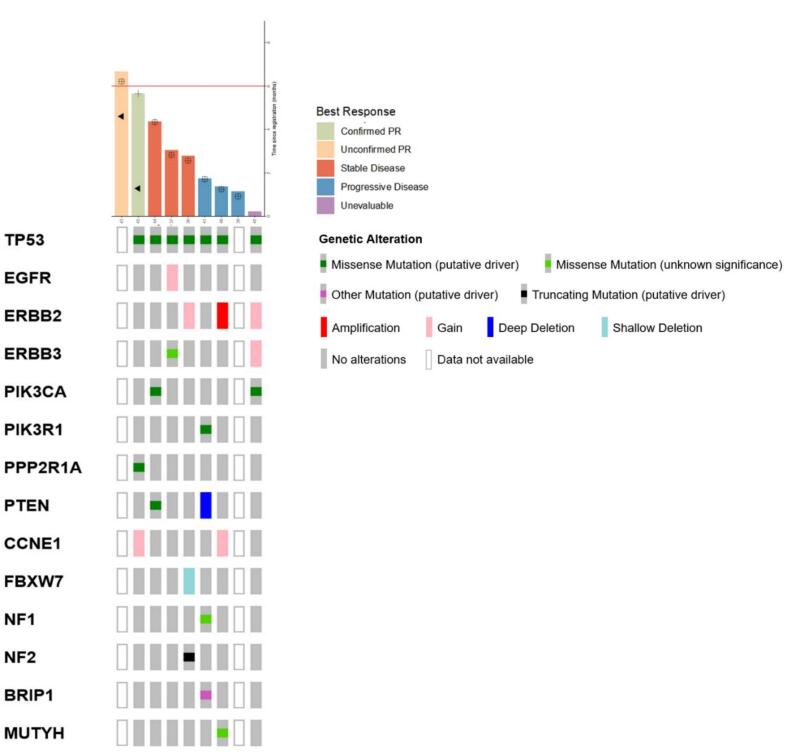
Selected molecular alterations of interest in enrolled patients.

Molecular alterations observed in the tumors of patients enrolled in the trial are shown in Fig. 2. The small sample size limited any formal analyses. Consistent with the eligibility criteria, *TP53* mutations were detected in all tumors. Of the two patients who experienced the longest clinical benefit, molecular data was not available on one patient’s sample. For the second patient, who experienced a confirmed response, the tumor had evidence of a mutation in *PPP2R1A* and low copy number gain of *CCNE1* (estimated <6 copies).

## 4 Discussion

In this single-arm open-label phase II trial, Wee1 inhibition with adavosertib in 9 patients with *TP53*-mutated UCS had limited clinical activity, with objective responses noted in 2 patients (22.2 %) with 1 patient remaining on treatment for over 6 months, and an additional 3 patients with initial stable disease at first study assessment. Treatment-related AEs were consistent with the expected adverse effect profile as seen in prior studies (Liu et al., 2021, Leijen et al., 2016, Cuneo et al., 2019). As observed with other trials, a majority of patients receiving adavosertib required dose modification, with 8 of 9 patients requiring a dose hold and 5 of 9 patients requiring at least one dose reduction.

**Fig. 3:**
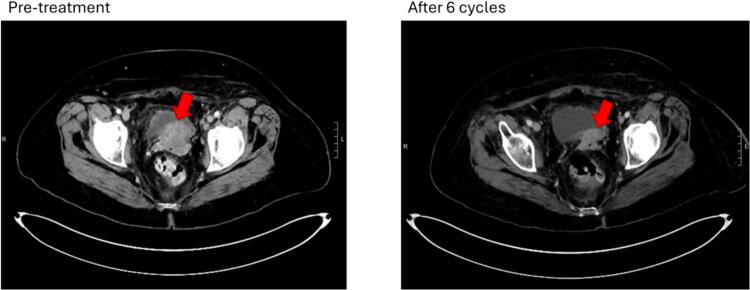
Radiographic response in patient with four prior lines of therapy. This patient had previously received carboplatin and paclitaxel, followed by weekly paclitaxel, lenvatinib and pembrolizumab, and single agent bevacizumab. Red arrow demonstrates significant decrease in volume of a vaginal cuff tumor.

While adavosertib showed limited overall activity, interpretation of these results is limited given the small number of patients enrolled due to early study termination based upon the pharmaceutical collaborator’s decision to halt development of adavosertib. Discontinuation of continued study of adavosertib by the sponsor was due to the narrow therapeutic window and relatively high rate of TRAEs. Two patients (22.2 %) did have evidence of disease response with 1 patient remaining on treatment for over 6 months. Three patients (33.3 %) had stable disease with time on treatment in this historically difficult to treat population. One patient had significant decrease in disease burden despite progression on four prior lines of therapy, including carboplatin/paclitaxel and lenvatinib/pembrolizumab (Fig. 3). Additionally, dose modifications and dose holds may have affected the therapeutic efficacy of adavosertib. In the ADAGIO study of adavosertib in uterine serous cancers, the narrow therapeutic window was noted as a potential factor limiting durable efficacy of the drug (Liu et al., 2021, Liu et al., 2023). Other Wee1 targeting agents are in development; the most advanced is azenosertib, which has reported increased selectivity for Wee1 and has reported preliminary signals of activity in uterine serous cancer (NCT04814108) and platinum-resistant ovarian cancer (NCT05128825), with phase 2 trials ongoing (Funda Meric-Bernstam et al., 2022, Huang et al., 2021). Additional trials of azenosertib have evaluated it in combination with chemotherapy with reported activity in ovarian cancer (Liu et al., 2023) and ongoing trials are evaluating its activity in platinum-resistant ovarian cancer (NCT04516447).

Because of the small study size and limited observed clinical activity, biomarker analyses were not feasible in this study. Additionally, the patient who experienced the best clinical response to adavosertib unfortunately did not have next generation sequencing available on her tissue for analysis. The patient who experienced a confirmed response did have evidence of both *PPP2R1A* mutation and low copy number gain of *CCNE1*. *PPP2R1A* mutations have been separately described as potentially conveying sensitivity to inhibition of PKMYT1, which is a kinase with some parallel functions to Wee1 (Yap et al., 2023). *CCNE1* amplification and high Cyclin E protein expression have also been described as possible biomarkers for Wee1 inhibitor sensitivity (Fu et al., 2023). However, although both *PPP2R1A* and *CCNE1* alterations may increase replication stress and susceptibility to adavaosertib, the single sample available this trial precludes definitive conclusions about whether these alterations were contributory to the response observed.

Since the initiation of this study several studies have evaluated recurrent endometrial cancer with some encouraging potential advances in recurrent UCS therapeutic options. The most exciting results are from the STATICE trial which evaluated the antibody-drug conjugate trastuzumab deruxtecan (T-DXd) in 32 patients with advanced or recurrent UCS expressing human epidermal growth factor recptor-2 (HER-2). The results of this study were highly encouraging, with a reported ORR of 54.5 % in HER2 high (IHC 2/3+) and 70.0 % in HER2 (IHC 1+) low tumors (Nishikawa et al., 2023). More recently, the FDA granted accelerated approval of trastuzumab deruxtecan in HER2-high (IHC 3+) tumors in a disease-agnostic manner based upon the results of the DESTINY PanTumor02 trial, and this is therefore a therapeutic option for select patients with UCS.

Other potential therapeutic options have been considered including immunotherapy combinations and hormonal therapy have yielded modest responses in UCS. Though the FDA approval for pembrolizumab and lenvatinib in mismatch repair-proficient endometrial cancer does not limit the use of this regimen to specific histologic subtypes, it is worthwhile to note in the KEYNOTE-775 study on which this approval is based on excluded patients with UCS. The activity of lenvatinib/pembrolizumab in recurrent UCS is unclear; one small single-institution study of cases found no ORR with using this combination in UCS cases, while a study examining a lower starting dose of lenvatinib reported limited activity, with 3 of 12 patients with UCS experiencing a response (Makker et al., 2022, Hunt et al., 2021). Hormonal therapies have also been studied in UCS, with the PARAGON trial (ANZGOG 0903) prospectively evaluating anastrazole with ER and/or PR positive leiomyosarcoma or UCS patients. In the 7 patients with UCS, the clinical benefit rate at 3 months was 43 % with a median duration of clinical benefit of 5.6 months; all 7 patients had stable disease but no objective responses were observed, and median PFS was 2.7 months.

The limited activity apart from T-DXd of other therapies in UCS highlights the importance of developing further therapies or combinatorial strategies in this space. While we saw some limited signals of activity from adavosertib monotherapy in the small number of patients on this study, further understanding of the biology of UCS may guide future approaches to leverage Wee1- or other DNA damage response-directed therapies and allow selection of individual patients most likely to benefit. One study using whole exome sequencing found that 25 % of UCS tumors exhibited the homologous recombination deficiency signature Signature-3, and that UCS cell lines harboring a dominant Signature-3 were associated with increased sensitivity to olaparib (Tymon-Rosario et al., 2022). Combined therapy with PARP inhibitors and Wee1 inhibitors have been previously shown to induce replication stress, DNA damage, and checkpoint damage, with evidence of potential synergy in preclinical models of breast and ovarian cancer (Fang et al., 2019). Studies combining Wee1 inhibitors and PARP inhibitors are being explored in ovarian cancer (NCT05198804); however the overlapping hematologic toxicities of these two agents may limit combinatorial dosing (Serra et al., 2022). Another combinatorial partner of interest may be the ataxia telangectasia and Rad3-related (ATR) inhibitors (Gourley et al., 2019, Manavella et al., 2023). *In vitro* and *in vivo* studies have reported sensitivity of UCS cell lines to the ATR inhibitor elimusertib, and a separate study has reported synergy between Wee1 and ATR inhibition in *CCNE1-*amplified ovarian and endometrial cancer models (Manavella et al., 2023). Where *CCNE1* amplification is common in UCS, the combined activity of Wee1 and ATR inhibition may be of interest in this population. However, similar to the Wee1/PARP inhibitor combination, overlapping hematologic AEs may require exploration of alternative dosing schedules such as sequential dosing. *In vitro* and *in vivo* models demonstrate possible rationale for combining T-Dxd with Wee1 inhibition in Her-2 expressing tumors with co-occurring CCNE1 amplification as this has been implicated in resistance to trastuzumab therapy; here again, overlapping hematologic AEs would need to be considered (DiPeri et al., 2023).

In this study, adavosertib showed limited activity in a heavily pre-treated unselected population of patients with recurrent UCS. Our results suggest that Wee1 inhibition monotherapy may impart toxicity with limited clinical benefit in UCS; however, given the molecular alterations common in UCS and potential for underlying high replication stress in these tumors, future targeted combinatorial strategies may still be of interest in these tumors with currently very limited treatment options. Biomarkers to identify patients who have the greatest benefit from Wee1 inhibitors or Wee1 inhibitor combinations are also needed and may help to direct future therapeutic development.

## CRediT authorship contribution statement

**Stephanie Cham:** Writing – original draft, Project administration, Investigation, Conceptualization. **Niya Xiong:** Writing – review & editing, Visualization, Formal analysis, Data curation. **Nabihah Tayob:** Visualization, Validation, Formal analysis. **Carolyn Krasner:** Writing – review & editing, Project administration. **Alexi A. Wright:** Writing – review & editing, Project administration. **Elizabeth K. Lee:** Writing – review & editing, Project administration. **Hannah Sawyer:** Project administration, Data curation. **Cara Mathews:** Writing – review & editing, Project administration. **Panagiotis A. Konstantinopoulos:** Writing – review & editing, Project administration. **Ursula A. Matulonis:** Writing – review & editing, Project administration. **Joyce F. Liu:** Writing – review & editing, Validation, Supervision, Project administration, Investigation, Conceptualization.

## Declaration of competing interest

The authors declare that they have no known competing financial interests or personal relationships that could have appeared to influence the work reported in this paper.
