# Supplementary material for: Phase 2 study of Wee1 inhibitor adavosertib in recurrent uterine carcinosarcoma
Source: Gynecol Oncol Rep. 2025 Jun 24;60:101796. doi: 10.1016/j.gore.2025.101796 (PMC12269869; doi:10.1016/j.gore.2025.101796)
Supplement: Supplementary data 1 [file mmc1.docx]

Supplemental Table 1. All treatment-emergent adverse events

| **Adverse Events** | **Grade 1** | **Grade 2** | **Grade 3** | **Grade 4** | **All Grade** |
| --- | --- | --- | --- | --- | --- |
| Any | 0(0%) | 2(22%) | 5(56%) | 2(22%) | 9(100%) |
| Fatigue | 0(0%) | 4(44%) | 3(33%) | 0(0%) | 7(77%) |
| Diarrhea | 1(11%) | 3(33%) | 3(33%) | 0(0%) | 7(77%) |
| Nausea | 2(22%) | 4(44%) | 1(11%) | 0(0%) | 7(77%) |
| Abdominal pain | 2(22%) | 2(22%) | 1(11%) | 0(0%) | 5(55%) |
| Creatinine increased | 5(56%) | 0(0%) | 0(0%) | 0(0%) | 5(56%) |
| Anemia | 0(0%) | 2(22%) | 2(22%) | 0(0%) | 4(44%) |
| Vomiting | 3(33%) | 1(11%) | 0(0%) | 0(0%) | 4(44%) |
| Platelet count decreased | 1(11%) | 0(0%) | 1(11%) | 1(11%) | 3(33%) |
| Neutrophil count decreased | 1(11%) | 1(11%) | 0(0%) | 1(11%) | 3(33%) |
| Pain | 0(0%) | 2(22%) | 1(11%) | 0(0%) | 3(33%) |
| Hyponatremia | 2(22%) | 0(0%) | 1(11%) | 0(0%) | 3(33%) |
| Generalized muscle weakness | 2(22%) | 0(0%) | 1(11%) | 0(0%) | 3(33%) |
| Fall | 1(11%) | 2(22%) | 0(0%) | 0(0%) | 3(33%) |
| Dyspnea | 1(11%) | 2(22%) | 0(0%) | 0(0%) | 3(33%) |
| Anorexia | 2(22%) | 1(11%) | 0(0%) | 0(0%) | 3(33%) |
| Dizziness | 2(22%) | 1(11%) | 0(0%) | 0(0%) | 3(33%) |
| Aspartate aminotransferase increased | 3(33%) | 0(0%) | 0(0%) | 0(0%) | 3(33%) |
| Thromboembolic event | 0(0%) | 1(11%) | 1(11%) | 0(0%) | 2(22%) |
| Insomnia | 0(0%) | 2(22%) | 0(0%) | 0(0%) | 2(22%) |
| Gastrointestinal disorders - Other, specify | 1(11%) | 1(11%) | 0(0%) | 0(0%) | 2(22%) |
| Fever | 1(11%) | 1(11%) | 0(0%) | 0(0%) | 2(22%) |
| Sinus tachycardia | 2(22%) | 0(0%) | 0(0%) | 0(0%) | 2(22%) |
| Edema limbs | 2(22%) | 0(0%) | 0(0%) | 0(0%) | 2(22%) |
| Hypomagnesemia | 2(22%) | 0(0%) | 0(0%) | 0(0%) | 2(22%) |
| Back pain | 2(22%) | 0(0%) | 0(0%) | 0(0%) | 2(22%) |
| Ascites | 0(0%) | 0(0%) | 1(11%) | 0(0%) | 1(11%) |
| Small intestinal obstruction | 0(0%) | 0(0%) | 1(11%) | 0(0%) | 1(11%) |
| Urinary tract infection | 0(0%) | 0(0%) | 1(11%) | 0(0%) | 1(11%) |
| Confusion | 0(0%) | 0(0%) | 1(11%) | 0(0%) | 1(11%) |
| Hypotension | 0(0%) | 0(0%) | 1(11%) | 0(0%) | 1(11%) |
| Blurred vision | 0(0%) | 1(11%) | 0(0%) | 0(0%) | 1(11%) |
| Dry eye | 0(0%) | 1(11%) | 0(0%) | 0(0%) | 1(11%) |
| Floaters | 0(0%) | 1(11%) | 0(0%) | 0(0%) | 1(11%) |
| Constipation | 0(0%) | 1(11%) | 0(0%) | 0(0%) | 1(11%) |
| Alanine aminotransferase increased | 0(0%) | 1(11%) | 0(0%) | 0(0%) | 1(11%) |
| Dehydration | 0(0%) | 1(11%) | 0(0%) | 0(0%) | 1(11%) |
| Hypoalbuminemia | 0(0%) | 1(11%) | 0(0%) | 0(0%) | 1(11%) |
| Acute kidney injury | 0(0%) | 1(11%) | 0(0%) | 0(0%) | 1(11%) |
| Pleural effusion | 0(0%) | 1(11%) | 0(0%) | 0(0%) | 1(11%) |
| Hot flashes | 0(0%) | 1(11%) | 0(0%) | 0(0%) | 1(11%) |
| Bloating | 1(11%) | 0(0%) | 0(0%) | 0(0%) | 1(11%) |
| Gastroesophageal reflux disease | 1(11%) | 0(0%) | 0(0%) | 0(0%) | 1(11%) |
| Oral pain | 1(11%) | 0(0%) | 0(0%) | 0(0%) | 1(11%) |
| Gait disturbance | 1(11%) | 0(0%) | 0(0%) | 0(0%) | 1(11%) |
| Localized edema | 1(11%) | 0(0%) | 0(0%) | 0(0%) | 1(11%) |
| Non-cardiac chest pain | 1(11%) | 0(0%) | 0(0%) | 0(0%) | 1(11%) |
| Alkaline phosphatase increased | 1(11%) | 0(0%) | 0(0%) | 0(0%) | 1(11%) |
| Blood bilirubin increased | 1(11%) | 0(0%) | 0(0%) | 0(0%) | 1(11%) |
| Flank pain | 1(11%) | 0(0%) | 0(0%) | 0(0%) | 1(11%) |
| Pain in extremity | 1(11%) | 0(0%) | 0(0%) | 0(0%) | 1(11%) |
| Dysgeusia | 1(11%) | 0(0%) | 0(0%) | 0(0%) | 1(11%) |
| Depression | 1(11%) | 0(0%) | 0(0%) | 0(0%) | 1(11%) |
| Hematuria | 1(11%) | 0(0%) | 0(0%) | 0(0%) | 1(11%) |
